# A cell atlas to guide research into appetite control

**DOI:** 10.7554/eLife.108863

**Published:** 2025-09-03

**Authors:** Greta Lommi, Thomas A Lutz

**Affiliations:** 1 Institute of Veterinary Physiology, University of Zurich Zurich Switzerland

**Keywords:** dorsal vagal complex, appetite, area postrema, nucleus of the solitary tract, Mouse, Rat

## Abstract

An atlas of all the cell types in the dorsal vagal complex of rodents will help neuroscientists seeking to understand appetite and researchers working to design better drugs for the treatment of obesity and related disorders.

**Related research article** Hes C, Tomlinson AJ, Michielsen L, Murdoch HJ, Soltani F, Kokoeva M, Sabatini PV. 2025. A unified rodent atlas reveals the cellular complexity and evolutionary divergence of the dorsal vagal complex. *eLife* **14**:RP106217. doi: 10.7554/eLife.106217.

Have you ever wondered what makes one neuron different from another? For example, why do some neurons send the signal to your brain that it’s time to eat, while others tell your brain that you feel full and don’t need to eat any more? Neuroscientists have long been fascinated by these questions, searching for the features that define each neuron and shape its role in the brain.

When it comes to appetite and eating, researchers focus on a region of the brain called the dorsal vagal complex (DVC) – a small but powerful hub in the caudal brainstem that helps control energy balance (Grill and Hayes, 2012; Gautron et al., 2015; Cheng et al., 2021). In particular, the DVC and its neurons have emerged as promising therapeutic targets for treating conditions ranging from obesity (Yanovski and Yanovski, 2014) to cancer anorexia-cachexia syndrome (Borner et al., 2018).

Yet, despite its importance, the full range of cell types in the DVC – and how they compare across species – remains somewhat unknown. A comprehensive atlas of the cells in the DVC would be of enormous help to researchers trying to understand these cells, their function and their potential as therapeutic targets. And given that much of this research begins with experiments on rodents, it would be particularly helpful to know about differences between the DVC in mice and rats.

Now, in eLife, Paul Sabatini of McGill University and colleagues – including Cecilia Hes as first author – report that they have used a technique called single-nuclei RNA sequencing to conduct an unprecedented molecular census of all the cells, neuronal and non-neuronal, in the DVC of mice and rats to produce such an atlas (Hes et al., 2025). A large number of cell types are found in both mice and rats, but some are only found in mice, and others are only found in rats (Figure 1), underscoring the cellular complexity and heterogeneity of the DVC.

**Figure 1.**
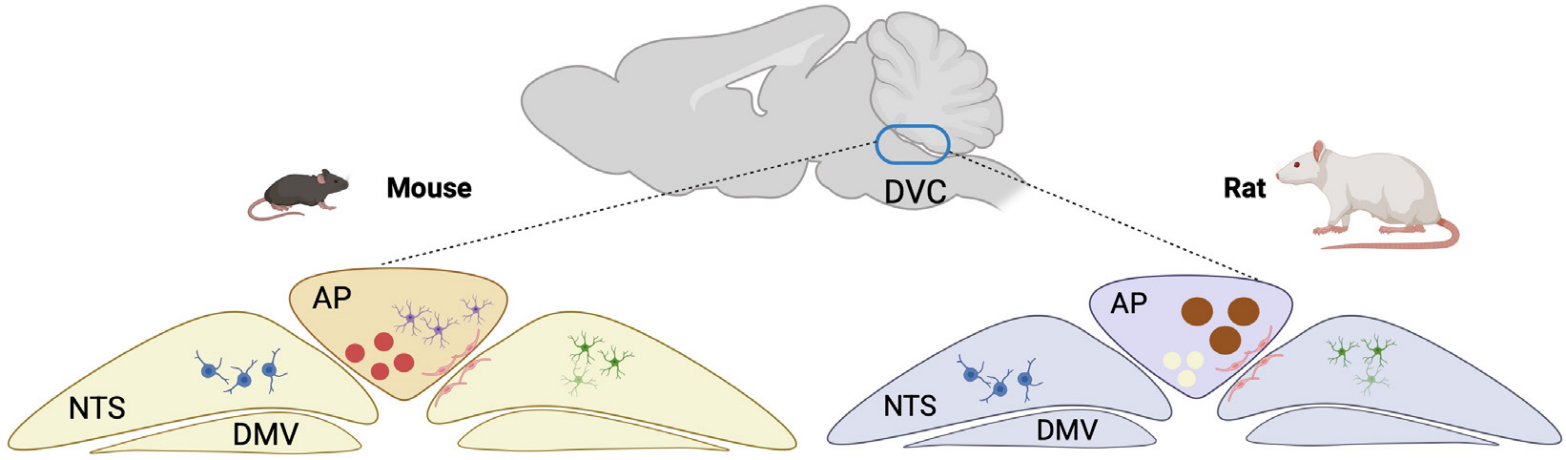
Species-specific cell types in the DVC of mice and rats. A schematic of the rodent brain. The dorsal vagal complex (DVC; blue oval) is composed of three areas: the area postrema (AP), which is closely connected to the bloodstream; the nucleus of the solitary tract (NTS), which sends signals to the brain, based on information it has received from the gastrointestinal tract; and the dorsal motor nucleus of the vagus (DMV), which is a major part of parasympathetic nervous system neurons. Some cell types – such as oligodendrocytes (blue), astrocytes (green), and endothelial cells (pink) – are found in both mice and rats. However, calcium-permeable astrocytes (lilac) and neurons that express the hormone GLP-1 (red) are only found in mice (left), while immunity-akin neurons (yellow) and ortus-akin neurons (which express the leptin receptor; brown), are only found in rats (right). Created with BioRender.com.

A major strength of this work is the way that Hes et al. – who are based at McGill, the University of Michigan and Leiden University – combined their new data with results from two earlier studies of mice (Ludwig et al., 2021; Dowsett et al., 2021). This resulted in a sample of over 170,000 cells, which they carefully checked to ensure the cell types used to classify the cells were consistent across all datasets.

The researchers also analysed over 12,000 cells from rats, grouped them into cell types, and matched these cell types to their mouse counterparts using the five most characteristic genes for each population. Some of these cell types had not been seen before. In the end, based on an analysis of more than 180,000 cells, Hes et al. were able to identify 123 different cell types.

Beyond cataloguing cell types, the study explored functional responses to feeding. Drugs targeting the DVC, such as amylin (Kruse et al., 2021) and GLP-1 mimetics (Knudsen and Lau, 2019), cause meaningful weight loss, but are often accompanied by gastrointestinal side effects (Wadden et al., 2021; Wilding et al., 2021). Since the DVC cells that control appetite and nausea are separate (Huang et al., 2024), identifying specific cell populations will help researchers to identify safer therapies. Hes et al. also examined neurons in mice that respond transcriptionally to eating after fasting, and found that nearly all neuron types exhibited changes in gene expression, though the magnitude of many of these changes was small. This suggests that meal-related transcription alone may not provide precise targets for pharmacological intervention.

Despite its strengths, the study has some limitations. The atlas includes only mouse and rat data, limiting direct translation to humans or other species. The rat DVC atlas, in particular, was generated from just two animals, raising the possibility that some observed cell populations or gene expression patterns reflect individual variation rather than species-wide features. Additionally, single-nuclei RNA sequencing captures only nuclear transcripts, potentially missing cytoplasmic or low-abundance RNAs, and functional validation of the identified cell types is still needed to confirm their roles in appetite regulation. Finally, while transcriptionally detailed, the atlas would benefit from complementary spatial mapping to better contextualize anatomical organization.

That said, the atlas developed by Hes et al. lays the groundwork for future studies to zoom in on specific neuronal and non-neuronal populations. With this information, researchers can begin to ask how these cells control feeding and energy balance, and how they might be targeted to treat obesity and related disorders.
